# Spatial‐temporal distribution and sequence diversity of group a human respiratory syncytial viruses in Kenya preceding the emergence of ON1 genotype

**DOI:** 10.1111/irv.12948

**Published:** 2021-12-27

**Authors:** Julia Wangui, D. James Nokes, Victor A. Mobegi, James R. Otieno, Charles N. Agoti, Joseph J.N. Ngeranwa, Wallace D. Bulimo

**Affiliations:** ^1^ Department of Biochemistry Kenyatta University Nairobi Kenya; ^2^ Center for Virus Research, Kenya Medical Research Institute (KEMRI) Nairobi Kenya; ^3^ Department of Epidemiology and Demography, Kenya Medical Research Institute (KEMRI) ‐ Wellcome Trust Research Programme Kilifi Kenya; ^4^ School of Life Sciences and Zeeman Institute for Systems Biology and Infectious Disease Epidemiology Research (SBIDER) University of Warwick Coventry UK; ^5^ Department of Biochemistry University of Nairobi Nairobi Kenya; ^6^ Present address: Division of International Epidemiology and Population Studies, Fogarty International Center National Institutes of Health Bethesda Maryland USA

**Keywords:** genotypes, haplotypes, HRSVA, regions

## Abstract

**Background:**

Human respiratory syncytial virus (HRSV) is a major cause of severe viral acute respiratory illness and contributes significantly to severe pneumonia cases in Africa. Little is known about its spatial–temporal distribution as defined by its genetic diversity.

**Methods:**

A retrospective study conducted utilizing archived nasopharyngeal specimens from patients attending outpatient clinics in hospitals located in five demographically and climatically distinct regions of Kenya; Coast, Western, Highlands, Eastern and Nairobi. The viral total RNA was extracted and tested using multiplex real time RT‐PCR (reverse transcriptase polymerase chain reaction). A segment of the G‐gene was amplified using one‐step RT‐PCR and sequenced by Sanger di‐deoxy method. Bayesian analysis of phylogeny was utilized and subsequently median joining methods for haplotype network reconstruction.

**Results:**

Three genotypes of HRSVA were detected; GA5 (14.0%), GA2 (33.1%), and NA1 (52.9%). HRSVA prevalence varied by location from 33% to 13.2% in the Highlands and the Eastern regions respectively. The mean nucleotide diversity (Pi[π]) varied by genotype: highest of 0.018 for GA5 and lowest of 0.005 for NA1. A total of 58 haplotypes were identified (GA5 10; GA2 20; NA1 28). These haplotypes were introduced into the population locally by single haplotypes and additional subsidiary seeds amongst the GA2 and the NA1 haplotypes.

**Conclusions:**

HRSVA was found across all the regions throughout the study period and comprised three genotypes; GA5, GA2, and NA1 genotypes. The genotypes were disproportionately distributed across the regions with GA5 gradually increasing toward the Western zones and decreasing toward the Eastern zones of the country.

## 1 INTRODUCTION

Human respiratory syncytial virus (HRSV) is a major cause of bronchiolitis and pneumonia in children and infants. It is estimated to cause over 3 million hospitalizations and over 50,000 deaths in children below 5 years of age each year.^1^ Most individuals are infected during the first 2 years of life and re‐infections occur throughout lifetime.^2^, ^3^, ^4^, ^5^

HRSV is characteristically seasonal with highest incidence seen during the winter months in temperate countries and during the rainy seasons in the tropics.^6^ In Kenya, increased infections are seen in the months of March to August and October to May with least infections in September.^7^, ^8^, ^9^

HRSV is an enveloped virus with a negative‐sense single stranded RNA genome of approximately 15 200 nucleotides. It consists of 10 genes encoding 11 proteins.^2^ Two of these are surface proteins: G (attachment) and F (fusion). The G‐gene consists of two hypervariable regions separated by a central conserved region, which contains four cysteine residues at positions 173, 176, 182, and 186.^5^, ^10^ The second hypervariable region has been utilized in several studies to characterize the HRSV into genotypes.^4^, ^11^, ^12^, ^13^ There are two genetically distinct HRSV groups; A and B.^13^ These groups are further categorized into genotypes. Fourteen genotypes of HRSVA, have been reported to date: GA1–GA7, SAA1, NA1–NA4 and ON1–ON2).^7^, ^14^, ^15^, ^16^, ^17^

Information on the distribution of the HRSVA genotypes across Kenya is scarce, and the inter‐relationship of these viruses across the regions of the country remains unknown. This study presents data on viruses detected in five regions across Kenya over a 4‐year calendar period. The rainy seasons in Kenya's extensive geographical region are from March–May (long rains) and October–December (short rains). The objectives of this study were to identify the HRSVA genotypes, describe spatial–temporal distribution and their genetic diversity, and hence gain insight into patterns of introduction into and spread within the population.

## 2 METHODS

### 2.1 Study design and population

This was a retrospective study utilizing archived nasopharyngeal (NP) specimens obtained from subjects enrolled in an influenza surveillance protocol by the Department of Emerging Infectious Diseases (DEID) of United States Army Medical Research Directorate–Kenya. The specimens were collected from consenting adult patients or parents consenting on behalf of their children, with influenza‐like illness (ILI) and attending the outpatient clinics in public hospitals across Kenya. Individuals were recruited if they were at least 2 months of age, presented within 72 h of onset of illness showing the following symptoms; a cough or sore throat and a fever of ≥38°C at the time of presentation.^18^, ^19^ The public hospitals (numbering 8) were part of the DEID surveillance network that had distinct geographic and demographic representation of Kenya.^19^ These sentinel centers were further clustered into five geographically distinct regions, that is, Coast, Eastern, Western, Highlands and Nairobi. Since increased cases of HRSV were observed in the months of March to August and October to May with least infections in September, we considered the HRSV season in Kenya to start in September of each year and end in August of the following year.^15^ The study period covered parts of 4 calendar year period (March 2007–February 2010), coinciding with mid HRSV season in the first and fourth years of the study. The sampling period was selected based on two reasons: (i) The time specimen collection commenced at the eight surveillance sites and (ii) the beginning of long rains when there is increased number of HRSV infections.

### 2.2 Ethical approval statement

This study was approved by the KEMRI Scientific and Ethical Review Unit (SSC # 2051) and the Walter Reed Army Institute of Research Institutional Review Board (WRAIR # 1267 sub‐protocol 1).

### 2.3 Study specimens and laboratory procedures

The NP swabs were collected in viral transport media (VTM) at the sentinel centers and transported to the country's National Influenza Center (NIC) for testing and storage.^20^ Total viral RNA was extracted from 2300 specimens using a commercial RNA extraction kit (QIAamp viral RNA mini kit®, Qiagen Inc., Valencia, CA, USA). The RNA was subsequently tested using modified multiplex real time reverse transcription Polymerase Chain Reaction.^21^

A segment of G‐gene was amplified using one‐step Reverse Transcriptase Polymerase Chain Reaction method (RT‐PCR) by utilizing the QIAGEN Ltd kit. Each reaction comprised of two primers; AG20, forward primer (5′‐GGGGCAAATGCAAACATGTCC‐3′) and F164, the reverse primer (5′‐GTTATGACACTGGTATACCAACC‐3′). These primers targeted the ectodomain region of the RSV G‐gene and part of the F gene.^22^ Thermocycling was performed at 50°C for 30 min (1 cycle), 95°C for 15 min (1 cycle), [94°C for 30 s, 54°C for 30 s, 72°C for 1 min] 40 cycles and 72°C for 10 min.^22^

Thereafter, the One‐Step RT‐PCR product was utilized in a 50‐μl reaction assay of the nested PCR method using the Qiagen Taqman PCR kit. This assay comprised of two primers; BG10 forward primer (5′‐GCAATGATAATCTCAACCTC‐3′) and FI reverse primer (5′‐CAACTCCATTGTTATTTGCC‐3′).^17^ Thermocycling conditions were at 95°C for 2 min (1 cycle), [95°C for 45 s, 54°C for 45 s, 72°C for 1 min] 30 cycles and an extension of 5 min at 72°C. The amplified products were visualized by electrophoresis on 2% agarose gel with ethidium bromide staining (approximately 650 bp). The amplicons were sequenced using Sanger dideoxy termination method utilizing two of the primers previously used in the nested PCR (BG10, F1). Additionally, two group specific primers; G523F (5′‐ATATGCAGCAACAATCCAAC‐3′) and G523R (5′‐GTTGGATTGTTGCTGCATAT‐3′) were incorporated.^22^

### 2.4 Sequence analysis

Raw sequence data was assembled using DNA baser version 3.2.^23^ The sequence contigs were manually edited and trimmed using BioEdit program version 7.2.5.^24^ Multiple sequence alignment was conducted using ClustalW algorithm in the BioEdit program^24^ and Muscle v3.8.31 software.^25^ These sequences were deposited in the GeneBank (accession numbers OK458563‐OK458679).

### 2.5 Phylogenetic analysis

The phylogenetic relationships were examined based on the nucleotide sequences in the full segment and the second hypervariable region of the G‐gene and were inferred using MrBayes software version 3.2.^13^, ^26^, ^27^ The tree was visualized using Fig tree v1.4.3 software.^28^ Since the phylogenetic clustering of the sequences in the two sets of analysis was similar, we are reporting the analysis based on the second hypervariable region (300 nucleotides) corresponding to 592–891 nucleotides of the prototype A2, accession number JX198138.^29^ To phylogenetically categorize our local sequences into their genotypes, 57 global sequences of known genotype designation were included.

### 2.6 Population analysis and network reconstruction

The entire sequence obtained from the Sanger dideoxy termination method (633 nucleotides) was utilized. The DnaSP version 5.10 was utilized to deduce the existing haplotypes based on single nucleotide polymorphism.^30^, ^31^ MEGA X was used to compute the genetic diversity of these viruses. The mean nucleotide diversity in each genotype (entire population) [Pi(π)^a^] and within each region by genotype [Pi(π)^b^] were computed.^32^ Median joining algorithm implemented in the Network version 5.0.0.3 software was utilized to calculate the parameters for reconstruction of the haplotype networks.^30^

## 3 RESULTS

### 3.1 Detection of HRSVA

We detected HRSVA in 159 (6.9%) samples, with varying proportions by region; 27.7% (44) for Highlands, 20.1% for Western, 19.5% for Coast, 18.2% for Nairobi and 14.5% for Eastern. The age range of the study population was 2 months to 47 years, while those with HRSVA infection ranged from 3 months to 6 years.

### 3.2 Phylogenetic analysis

Three of the fourteen known HRSVA genotypes were found in this population. These were GA2, GA5, and NA1 genotypes. Most of the viruses belonged to the NA1 genotype (64 sequences; 52.9%), followed by GA2 genotype (40 sequences; 33.1%) and the rest of GA5 genotype with 17 sequences (14%).

**FIGURE 1:**
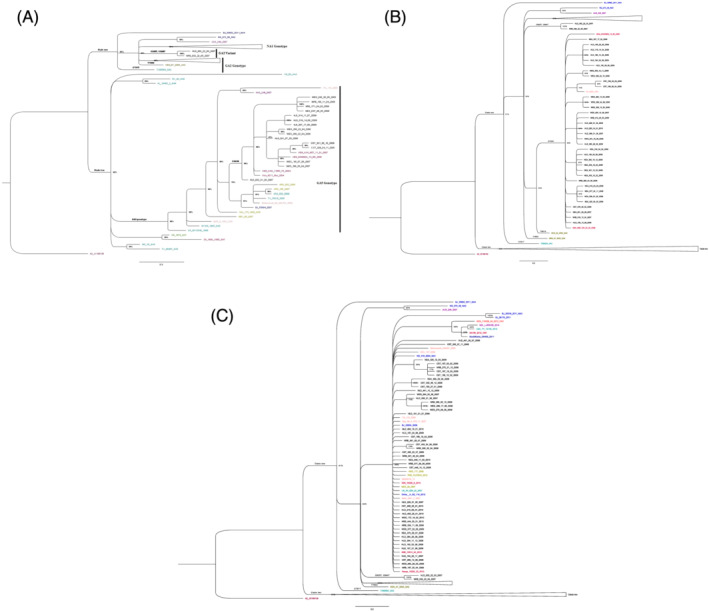
(A) Phylogenetic tree based on the 2nd hypervariable region of the G‐gene from samples collected from ILI presentations to eight health facilities across Kenya 2007–2010. This tree was constructed using the Bayesian method with a scale of 0.3 and the clade credibility of above 60%. Due to the large data size (178 sequences), we clustered our local sequences into their haplotypes (refer to methods) and obtained a representation of 97 of 121 local sequences for clear visibility on the phylogenetic tree. The tree was rooted on the prototype A2, accession number JX198138. Colored tips represent the global sequences accessed from Genbank. Clade two sequences belonging to genotypes GA3‐7 represented in detail while the branches in clade one has been collapsed (NA1‐4), GA2 and ON1 genotypes (see Figure 1B,C). (B) Clade one of the phylogenetic tree showing the GA2 genotype sequences. Colored tips belong to the global sequences. Among the GA2 sequences, there is an additional cluster (clade credibility of 100%) comprising of two sequences that were not previously identified. We categorized these sequences as variants of the GA2 genotype. (C) Clade one of the phylogenetic tree showing sequences in the NA1‐4 and ON1 genotypes. Colored tips comprise of the global sequences. All our local sequences were within the NA1 genotype

The local sequences were found on two major clades of the phylogenetic tree (Figure 1A); one clade comprised of the NA1 and GA2 genotypes while the other clade comprised of the GA5 genotype (clade two). The local GA5 sequences clustered away from those from elsewhere. These sequences were characterized by C643A resulting in an amino acid change (P215I) while those from elsewhere were characterized by P215L. Only one sequence from Mexico was similar to the prototype at this position.

The local GA2 sequences formed several clusters. Characteristically, these clusters comprised of sequences from the same region with only four remaining at the root of the clade (Figure 1B). In contrast, the majority of the NA1 sequences remained at the root of the clade with only fifteen of the local sequences (23%) forming additional clusters (Figure 1C). An additional cluster of two local sequences with clade credibility of 100% was observed on clade one (Figure 1B). These sequences were characterized by five nucleotide substitutions resulting in two amino acid changes; C600T, G673A (V225I), C684T, C821T, and C891G (R297K).

### 3.3 Spatial–temporal distribution of HRSVA genotypes across Kenya

Most of the viruses were from the Highlands region (33%) followed by the Western region (20%) while the least were from the Eastern region (Table 1). Overall, the genotype dominance varied across the five regions. GA5 genotype was predominant in the Western (47%) region, GA2 genotype (31%) in the Eastern region while NA1 genotype in three regions; Coast (24%), Nairobi (25%) and Highlands (34%).

**TABLE 1:** Distribution of HRSVA (121 sequences), illustrating the distribution per genotype (GA2, GA5, and NA1) and the number of haplotypes (Hap) by regions

Regions	*	GA2/Hap	GA5/Hap	NA1/Hap
Eastern	13.2 (16)	31/5	0/0	6/4
Coast	16.5 (20)	8/2	12/1	24/9
Highlands	33 (40)	**32**/7	29/3	**34**/9
Western	20 (24)	24/5	**47**/4	11/4
Nairobi	17.3 (21)	5/4	12/2	25/11

*Note*: *column presents the proportion of sequenced HRSVA by region (number of sequences). The dominant genotype in each region is in bold.

Further variation was observed in the distribution of the genotypes from season to season (Table 2). GA2 genotype was the most common genotype in season 1 while GA5 during season 2, and NA1 genotype in seasons 3 and 4. The GA5 genotype was not detected during season 4.

**TABLE 2:** Temporal distribution of sequenced positives (121) showing the number in each genotype (GA2, GA5, and NA1) per HRSV season covered in this study

Study period	*n*	GA2	GA5	NA1
Mar. 2007–Aug. 2007 (Season 1)	13	**7**	3	3
Sept. 2007–Aug. 2008 (Season 2)	30	10	**12**	8
Sept. 2008–Aug. 2009 (Season 3)	57	22	2	**33**
Sept. 2009–Feb. 2010 (Season 4)	21	1	0	**20**

*Note*: *n* column presents the number of sequenced HRSVA by the study period corresponding to the HRSV season. The GA2 variants were both observed during season 1 and are grouped in the GA2 category. In highlight (bold) is the leading genotype in each season.

### 3.4 The genetic diversity and haplotype analysis of HRSVA

The nucleotide mean distance within the GA2, GA5 and NA1 genotypes was 0.0085, 0.018 and 0.005 respectively. We further investigated the nucleotide mean distances per region and genotype and found that amongst the GA2 viruses, it was lowest in the Eastern region (0.005) and highest in the Western region (0.008). Amongst the NA1 viruses, it was lowest in Nairobi and Western regions (0.003) while Coast and Eastern regions had the highest (0.008). Amongst the GA5 viruses, there was an absence of diversity on the Coast region (nucleotide mean distance of 0.00), and highest nucleotide mean distance in the Western region (0.017) (Table 3).

**TABLE 3:** The group mean distances of the sequenced positives of each genotype by regions across Kenya

Region	GA2*genotype	GA5 genotype	NA1 genotype
Eastern	0.005	NA	**0.008**
Coast	0.007	0.00	**0.008**
Highlands	0.006	0.007	0.004
Western	**0.008**	**0.017**	0.003
Nairobi	0.007	0.003	0.003

*Note*: *excludes the 2 GA2 variant sequences. NA indicates no sequences. GA5 genotype was not detected in the region.

Haplotype diversity across the genotypes ranged from 0.919 in the NA1 genotype to 0.952 in the GA2 genotype (Table 4).

**TABLE 4:** HRSVA haplotype and nucleotide diversity, by genotype, from sequences across Kenya: 2007–2010

Genotype	*N*	Hap/V	Hd	Pi (π)^a^/Pi (π)^b^
GA2	37^*^	17/19	0.926	0.009/0.007
GA5	16	10/22	0.933	0.018/0.007
NA1	62	28/39	0.919	0.005/0.005

*Note*: *N*: number of sequences (*excludes 2 GA2 variant sequences), Hap: the number of haplotypes, V: variable sites, Hd: haplotype diversity, Pi (π)^a^: mean nucleotide diversity for the entire population and Pi (π)^b^: mean nucleotide diversity within regions by genotype.

### 3.5 Spatial distribution of local HRSVA haplotypes per genotype

**FIGURE 2:**
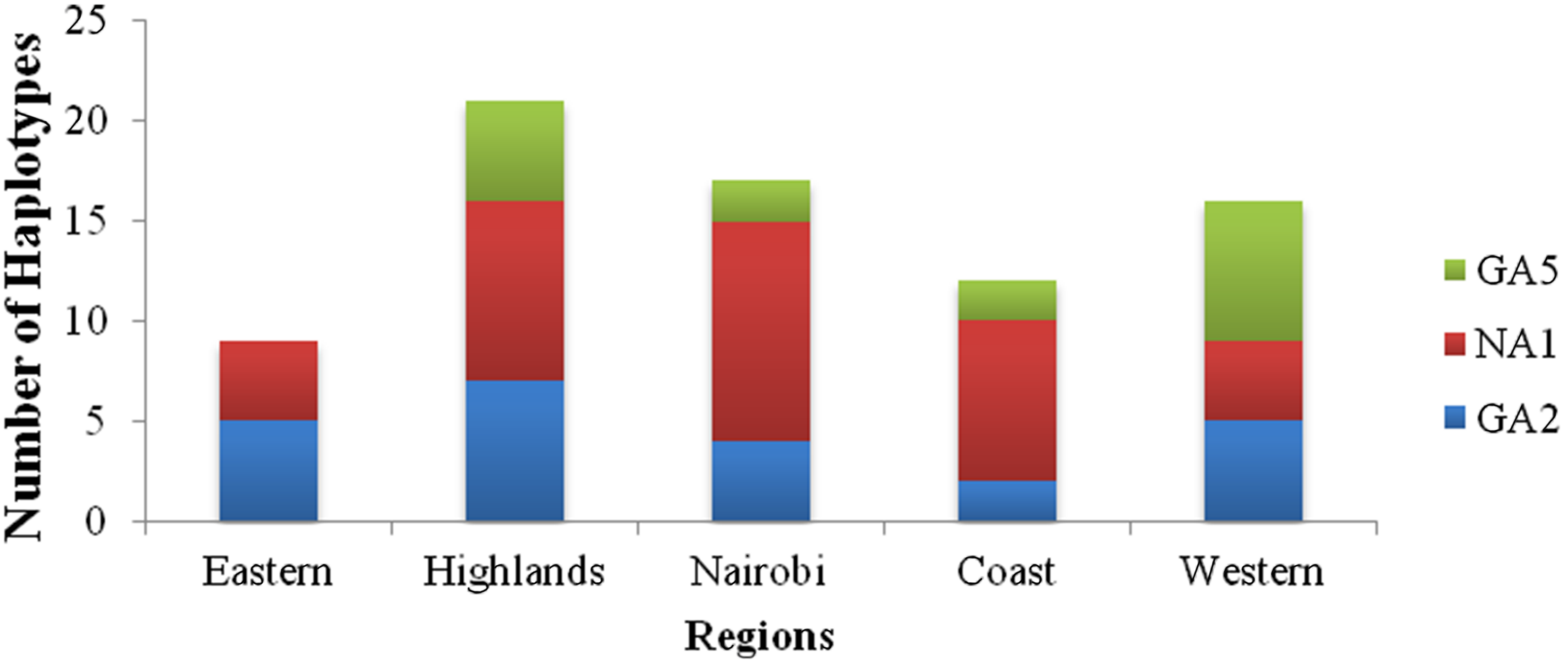
Spatial distribution of the local haplotypes: The graph represents the distribution of haplotypes in each region per genotype

The majority of the NA1 sequences were found in the Highlands region (Figure 2A); however, the Nairobi region had the highest number of haplotypes (Table 1) and the least in the Eastern and Western regions. The GA2 haplotypes were dominant in the Eastern region while the GA5 haplotypes dominated the Western region.

### 3.6 Inter‐relationship of the local HRSVA haplotypes in the five regions across

**FIGURE 3:**
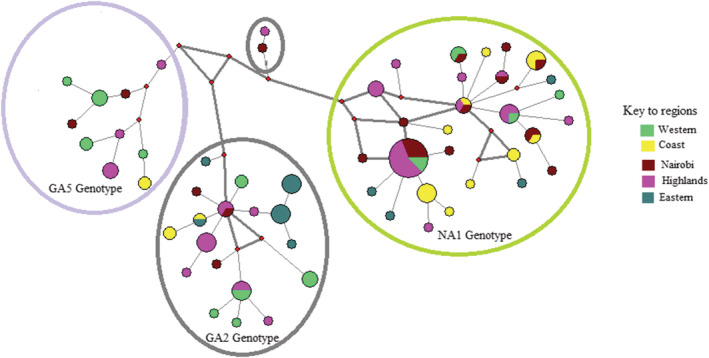
Spatial interaction of the Kenyan haplotypes identified through sentinel hospital outpatient surveillance from 2007–2010: Median‐joining network tree displaying the interrelationship of haplotypes per genotype across five regions. Each tree node (circle) represents a haplotype, color coded as per the region where it was detected. A multi‐colored node represents a haplotype found in multiple regions. Node size is directly proportional to the number of viruses in each haplotype. The tree displays haplotype interlinkage within and across regional boundaries. The branch length between haplotypes is not proportional to the number of mutations detected between the haplotypes. The four‐color coded circles represent the genotypes. The two haplotypes in the smaller circle belong to the GA2 variant viruses

We further explored the interrelationship and divergence of the local haplotypes as defined in the three genotypes across the five regions (Figure 3). These haplotypes were widely dispersed across the regions and a few were found in multiple regions.

All the GA5 haplotypes were unique to single regions while 15% of GA2 and 25% of NA1 haplotypes were found in multiple regions. The network tree further displayed divergence of haplotypes across the regions.

### 3.7 Seeding of the Kenyan HRSVA viruses

**FIGURE 4:**
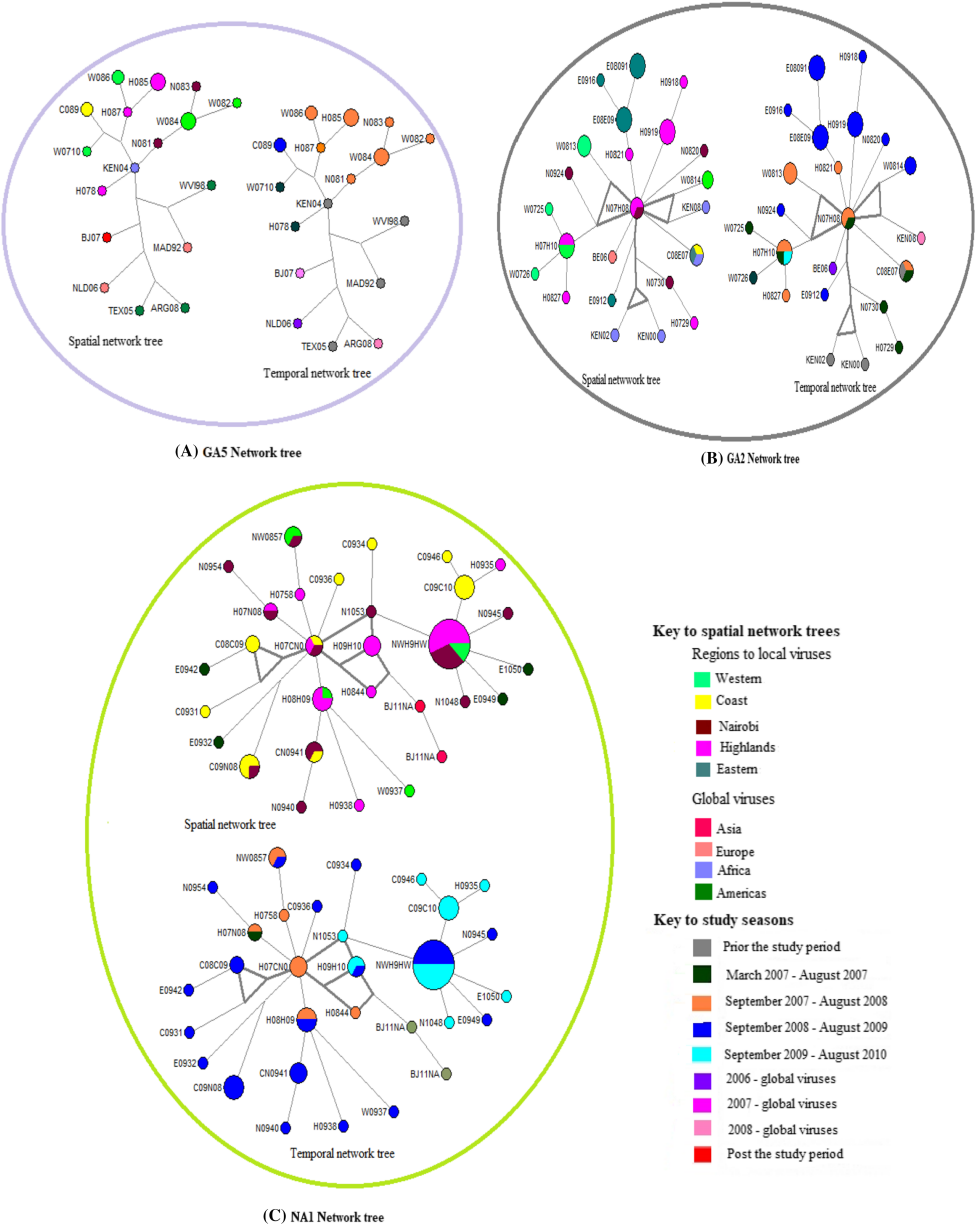
Seeding of haplotypes. These median joining networks illustrate the introduction patterns amongst 130 haplotypes. The color coding in the spatial network trees represent the regions for the haplotypes. The global haplotypes are as follows: Americas (ARGE08, TEXAS0, and WESTVI), Europe (NETHER, MAD92, and BE06), Asia (BJ07 and BJ11NA) and Africa (KEN04, KEN02, KEN08, and KEN00). The color‐coding in the temporal network trees represent the period prior, during and post the study. There are different color codes for the local and global haplotypes representing the four seasons of the study and the year of collection for the global haplotypes

Spatial–temporal clustering of haplotypes belonging to one hundred and thirty sequences (including thirteen global sequences) was utilized to investigate the seeding patterns of the local viruses. The network trees were reconstructed based on the circulating genotypes in the population as previously identified, that is, GA5, GA2, and NA1 (Figure 4).

The clustering of seventeen haplotypes found in the GA5 genotype illustrated a single seed amongst the local haplotypes (Figure 4A). The seed (KEN04 haplotype), was from a sequence collected in March 2004 from Kilifi, Kenya (accession number JQ838335). The GA2 genotype network tree comprised of local haplotypes and four global haplotypes (Figure 4B). Three of these were Kenyan sequences from a previous study (accession numbers AY524649, AY660672, and KF156387) while the fourth haplotype was from Belgium (accession number JX645860). This network tree displayed two possible seeds. The main seed, haplotype N07H08, was detected in Nairobi and Highlands regions during the first and the second seasons respectively. It seeded haplotypes from all regions including the Belgium haplotype. The subsidiary seed, haplotype H07H10, was detected in the Highlands region during the first and fourth seasons and in the Western region during the second season. It only seeded three haplotypes from the Highlands and Western regions. Of the local haplotypes, only one haplotype (E0912) and the GA2 variants (H0729 and H0730) that were not directly linked of these seeds.

Two local seeds were observed amongst the NA1 haplotype network tree (Figure 4C). The main seed, haplotype H07CN0, was detected in three regions during the second season. A subsidiary seed, haplotype NWH9HW, was also detected in three regions and comprised the majority of the viruses.

## 4 DISCUSSION

This study provides insight into the genetic diversity of the HRSVA viruses found in five regions of Kenya. These regions provide a temporal and demographic representation of the country covering the East, West, North and South (Nairobi, Western, Eastern, Coast and Highlands regions). The samples collected from March 2007 to February 2010 were randomly selected realizing 460 samples from each of the regions. Reports have indicated that HRSV distribution in Kenya varies between the Eastern and Western zones.^8^, ^33^

For the purposes of this report, we proposed that the HRSV season in Kenya starts in September of each year and ends in August of the following year. The phylogenetic analysis determined that there were only three genotypes in this population during this period: GA5, GA2 and NA1. The NA1 genotype was predominant while GA5 genotype the least dominant and was not detected in the Eastern region. These findings were similar to another local study conducted during the period coinciding with our study period which reported the GA5 (12.5%) to be the least dominant while GA2 genotype and its variants the most abundant (75%).^9^ However, when we examined the distribution of the genotypes by region we observed increased dominance of the GA5 genotype toward the Western region of the country. While this is true, the GA5 genotype was not detected during the fourth season of the study and the period coinciding with the season in other studies conducted locally.^9^

The phylogenetic tree displayed divergent clustering amongst the Kenyan genotypes. Amongst the GA5 viruses, our local sequences clustered away from the global sequences hence forming a distinctive clade. The leaves of this clade comprised of sub‐clusters with sequences mainly from the same regions thereby showing a tendency toward regional clustering. This is further supported by the low mean nucleotide diversity within regions and high mean nucleotide diversity for the entire GA5 population.

The clustering amongst the GA2 sequences was diverse hence forming three distinct clades; (i) comprised of global sequences obtained in Kenya prior the study period, (ii) comprised of our local sequences, and (iii) comprised of two of our local sequences that were different from the two categories mentioned above (i and ii). Due to the closeness of this clade phylogenetically with the GA2 genotype we considered this category as a variant of the GA2 genotype. Moreover, it was not detected after the first season. Overall, the GA2 sequences from the same season clustered together, an indication of temporal distribution pattern as reported elsewhere.^9^

The NA1 cluster was the largest, comprising of sixty‐four of our local sequences and additional global sequences. Some of these sequences had previously been identified as GA2 variants prior of the variant to a genotype.^16^ Unlike in the GA2 and the GA5 clusters, three quarters of sequences of this genotype remained at the root of the clade. This phenomenon is suggestive low diversity amongst these viruses. Only a quarter of these sequences formed additional clusters. An additional cluster comprising of global sequences of the ON1 genotype was also observed. This divergent clustering in the NA1 cluster supports the theory that the ON1 genotype was derived from NA1 genotype through a single duplication event.^34^ Although this genotype was first reported in December 2011 in Ontario, a retrospective study conducted in Panama from 2008 to 2012 found this genotype from a specimen (KF300973) collected in October 2010.^11^ This genotype was first detected in Kenya in February 2012.^7^ Our study, covering five regions encompassing one of the locations where this genotype was first detected, Coast region, did not find the ON1 genotype and therefore an indication that indeed, this genotype was introduced in the country after February 2010.

The highest prevalence of HRSVA was in the Highlands region. The distribution of the viruses varied from season to season. The GA2 and GA5 genotypes were predominantly found in the first and second seasons, respectively. This was followed by a decrease of the two genotypes occurring concurrently with a gradual increase of the NA1 genotype in the subsequent seasons. Consequently, the NA1 genotype became the predominant genotype (95%) at the end of the study period. These findings are consistent with those of a study conducted in Northern Kenya from September 2007 to March 2011 that reported the three genotypes and only two GA5 viruses.^9^ Our findings relate to other studies that have reported dominance of GA2 and NA1 genotypes coinciding with our study seasons: studies conducted in Belgium (2006–2011),^35^ Madrid (2007–2014), Panama (2010–2012),^5^ and another in Kenya covering thirteen seasons from 2000 to 2012.^9^ A study conducted in Brazil showed replacement of GA5 genotype with GA2 genotype during 2005–2008 seasons.^36^ This is an interesting shift in contrast to pre‐millennium reports in which the HRSVA genotypes were shown to co‐circulate with variation in genotype dominance and especially from mid 1970s–2000s.^4^, ^5^ The millennium studies have reported a shift from multiple genotype circulation to replacement of an existing genotype and its increased dominance.^5^ Locally, the viruses in the GA2 and NA1 genotypes were detected in all the five regions while the GA5 was only in the Coast, Nairobi, Highlands and Western regions.

Insight on how these viruses interrelated across the regions and seasons was gained through the median joining networks. The network trees showed coherent interrelationship patterns of the haplotypes circulating in the country and were distinctive for each genotype. The GA5 haplotypes had the highest haplotype diversity while the NA1 haplotypes had the least diversity with the highest number of variable sites. The GA5 haplotypes were unique to regions and seasons while GA2 and NA1 haplotypes were diverse with the minority being found in either of the following categories: (i) multiple regions concurrently during a single season, (ii) multiple regions simultaneously across the seasons, (iii) multiple regions during different seasons, (iv) unique to a single region across seasons, and (v) multiple regions in different seasons.

We further sought insight on the introduction patterns of our local HRSVA haplotypes. We observed unique patterns amongst the genotypes ranging from a single haplotype introduction to multiple introductions to seasons. Amongst the GA5 haplotypes, a single haplotype introduction was observed. This haplotype was from a previous study conducted at the Coast region prior to our study period. The GA2 haplotypes emerged from a local haplotype (N07H08), first detected in the Highlands and Nairobi regions during the first season. While it was the main source of introduction for the local haplotypes in this cluster, it was linked to a Belgium haplotype (BE06) which was detected in the period coinciding with our 1st season of the study period. Two subsidiary seeds were observed in this cluster. Uniquely, haplotypes from the Eastern region emerged from one of the subsidiary seeds (E08E09). Similarly, a single introduction was observed amongst the local NA1 haplotypes with additional subsidiary seeds. The introduction pattern in this cluster mainly illustrated temporal seeding patterns. A limitation to the study is that only parts of the first and last HRSV seasons (as defined in the Methods) had samples and hence caution should be taken in inferring to temporal patterns.

In conclusion, this study revealed three HRSVA genotypes circulating in the country during the study period. The genotypes were disproportionately distributed across the regions with GA5 increasingly populating toward the Western zones and decreasing toward the Eastern zones of the country. It further demonstrated diverse spatial and temporal interrelation of the Kenyan haplotypes across genotypes and establishes localized transmission across the regions.

## AUTHOR CONTRIBUTIONS

**Julia Wangui:** Conceptualization; data curation; formal analysis; methodology; project administration; writing. **D. James Nokes:** Conceptualization; formal analysis; funding acquisition; resources; software; supervision.  **Victor A. Mobegi:** Formal analysis; validation. **James R. Otieno:** Formal analysis; methodology; validation. **Charles N. Agoti:** Conceptualization; methodology; validation. **Joseph J.N. Ngeranwa:** Supervision. **Wallace D. Bulimo:** Conceptualization; formal analysis; funding acquisition; resources; supervision.

## CONFLICT OF INTEREST

The authors declare that they have no competing interests.

## DISCLAIMER

The views or insertions expressed herein are private views of the authors and are not to be construed to represent those of the US Department of Defense or Army.

### PEER REVIEW

The peer review history for this article is available at https://publons.com/publon/10.1111/irv.12948.

## Data Availability

The nucleotide sequences were submitted to the GeneBank and allocated accession numbers OK458563 ‐ OK458679.
